# Characterizing opioid agonist therapy uptake and factors associated with treatment retention among people with HIV in British Columbia, Canada

**DOI:** 10.1016/j.pmedr.2023.102305

**Published:** 2023-06-29

**Authors:** Kiana Yazdani, Katerina Dolguikh, Monica Ye, Jason Trigg, Ronald Joe, Scott D. Emerson, Julio S.G. Montaner, Rolando Barrios, Kate Salters

**Affiliations:** a British Columbia Centre for Excellence in HIV/AIDS, Vancouver, British Columbia, Canada; b The University of British Columbia, Vancouver, British Columbia, Canada; c Simon Fraser University, Burnaby, British Columbia, Canada; d Vancouver Coastal Health, Vancouver, British Columbia, Canada

**Keywords:** Opioid agonist therapy, Opioid use disorder, Treatment retention, HIV, AIDS, Administrative health data

## Abstract

Accidental overdoses are now the leading cause of death among people with HIV (PWH) in British Columbia (BC). We examined the utilization and retention of opioid agonist therapy (OAT).

Adult PWH (≥19 years) with ≥ 1 OAT dispensation in BC between 2008 and 2020 were included (n = 1,515). OAT treatment episodes were formed based on specific criteria for slow-release oral morphine (SROM), methadone, injectable OAT (iOAT), and buprenorphine/naloxone. Retention in treatment was defined as any episode lasting ≥ 12 months. Logistic regression with generalized estimating equations modeled retention-associated factors.

There was a 56.6% decline in OAT retention over time. Buprenorphine treatment exhibited significantly lower odds of retention (OR: 0.58; 95% CI: 0.36–0.92) compared to methadone. Conversely, no significant change in retention odds was observed for SROM (0.72; 0.33–1.54) and iOAT (0.81; 0.31–2.12). Factors associated with increased odds of retention included a 10-year increase in age (1.69; 1.46–1.95), previous retention history (1.96; 1.40–2.73), achieving OAT therapeutic dose (8.22; 6.67–10.14), and suppressed HIV viral load (1.35; 1.10–1.67). Individuals with a lifetime HCV diagnosis receiving iOAT were more likely to retain (3.61; 1.20–10.83). Each additional year on OAT during the study period was associated with a 4% increase in the odds of retention.

A significant proportion of PWH had a history of OAT prescribing but experienced low retention rates. Retention outcomes were more positive for SROM and iOAT. The association between OAT medication type and retention odds may be particularly influenced by HCV diagnosis. Optimal management of opioid use disorder among PWH, with an emphasis on attaining the therapeutic dose is crucial.

## Highlights

• There was a 56.6% decline in overall opioid agonist therapy (OAT) retention among people with HIV (PWH) between 2008 and 2020.
• Buprenorphine/naloxone was associated with reduced odds of retention.
• Conversely, the odds of retention in slow-release oral morphine (SROM) and injectable OAT (iOAT) did not change compared to methadone.
• Odds of retention were associated with a 3-fold increase among PWH with a lifetime diagnosis of hepatitis C virus (HCV) who were receiving iOAT.
• Factors found to be associated with significantly increased odds of retention include: achieving a therapeutic dose, increasing age, previous history of retention, suppressed HIV viral load, and increased number of days on OAT.

## 1 Introduction

Opioid use disorder (OUD) is a chronic condition characterized by the continuous use of opioids or synthetic opioids (e.g., heroin, fentanyl), associated with elevated rates of morbidity and mortality (Strang et al., 2020). In the past decade, the unprecedented rise in fatal overdoses has decreased life expectancy in British Columbia (BC), Canada (Ye et al., 2018). As of 2021, the province reported an all-time high rate of 43 overdose deaths per 100,000 deaths (British Columbia Coroners Service, 2019). Fentanyl, or other synthetic opioids, in combination with other drugs accounted for 86% of the deaths (British Columbia Coroners Service, 2019).

Opioid agonist therapy (OAT) is a safe method for treating OUD (Strang et al., 2020). Using medications to activate the opioid receptors, OAT reduces cravings for opioids and prevents withdrawal (Bell and Strang, 2020). Methadone, a long-acting synthetic opioid, and Buprenorphine/Naloxone (Suboxone®), a fixed-dose combination medication, are examples of key OAT products (Strang et al., 2020, Bell and Strang, 2020). Slow-release oral morphine (SROM/Kadian) and injectable OAT (iOAT) are emerging as increasingly popular alternatives for OAT and are particularly recommended for individuals with more severe OUD (Oviedo-Joekes et al., 2019, College, 2022).

There is robust evidence that OAT reduces all-cause and overdose-specific mortality among people with OUD (Sordo et al., 2017, Pearce et al., 2020, Santo et al., 2021). Furthermore, the individual- and community-level benefits of OAT are well-established (Academies et al., 2019, Volkow et al., 2014, Colledge-Frisby et al., 2022). However, barriers like strict program requirements, limited access, and stigma hinder treatment uptake and retention (Blanco and Volkow, 2019). A study in Vancouver, BC showed only half of the participants who initiated OAT between 2005 and 2018 were retained at any given time during the study period (Socías et al., 2020).

People with HIV (PWH) are disproportionately impacted by OUD and are at an elevated risk of dying from an overdose due to respiratory distress, liver impairment, or difficulty accessing OUD treatment (Green et al., 2012). In BC, the illicit drug toxicity has reduced survival gains achieved by combination antiretroviral therapy (ART) among PWH (St-Jean et al., 2021). Recent evidence indicates a shift in mortality patterns among PWH, with non-HIV causes, particularly accidental overdoses, becoming the leading cause of death from 2010 onwards (Yazdani et al., 2022, Salters et al., 2021).

Despite the promising outlook in HIV treatment, healthcare inequities among PWH with complex co-occurring comorbidities – such as OUD – are jeopardizing improved HIV outcomes (Salters et al., 2021). Studies have found that among people who inject drugs and have HIV, OAT independently influenced the HIV cascade of care and the odds of receiving ART were higher among lifetime recipients of OAT compared to non-OAT recipients (Mazhnaya et al., 2018, Mlunde et al., 2016). Contrary to the beneficial impact of OAT on HIV care, PWH are less likely to initiate timely OAT compared to patients without HIV who are similar in demographic characteristics (Wyse et al., 2019).

The study aimed to achieve three objectives: 1) identify and characterize PWH in BC who received OAT between January 2008 and March 2020; 2) assess the annual uptake of OAT and analyze trends in retention; and 3) identify factors associated with retaining individuals in OAT for twelve months or longer.

## 2 Methods

### 2.1 Data sources

The data for this research came from the Seek and Treat for Optimal Prevention of HIV/AIDS (STOP HIV/AIDS) study, which is an open bidirectional population-based longitudinal cohort held at the BC Centre for Excellence in HIV/AIDS (BC-CFE). This includes de-identified individual-level data on adults (age ≥ 19 years old) with HIV infection from 01 April 1996, to 31 March 2020. The study cohort was created by linking three provincial data sources: the BC-CFE Drug Treatment Program (DTP), the BC Centre for Disease Control (BCCDC) HIV/AIDS surveillance database (Centre, 2020), and datasets stewarded by Population Data BC, which houses data from various provincial administrative health databases. A detailed description of the STOP HIV/AIDS study has been provided previously (Heath et al., 2014, Nosyk et al., 2013).

In this study, we utilized data from several databases within the STOP HIV/AIDS cohort between 01 January 2008 and 31 March 2020. Demographic and laboratory information for all PWH were obtained from the DTP database (British Columbia Center for Excellence in HIV/AIDS, 2020). Comorbidities were identified using their respective International Classification Disease Codes, 9th revision, 9th revision clinical modification, and 10th revision, Canada (ICD-9/9-CM/10-CA) (Organization, 2019, World Health Organization, 2009) from two administrative databases: i) the Medical Services Plan Payment Information File (MSP), which includes all practitioner claims with one ICD-9/9-CM diagnostic code representing the reason for that visit (British Columbia Ministry of Health Medical Services Plan, 2016); ii) Discharge Abstract Database (DAD), which includes all acute hospitalizations in BC hospitals with up to 25 diagnostic codes (ICD-10-CA April 2001 onwards) per hospitalization, including one which represents the most responsible diagnosis for admission, and 20 procedure codes (Canadian Institute for Health Information, 2016). Information on all dispensed medications was retrieved from the PharmaNet database, according to Drug/Product Identification Number (DIN/PIN) (British Columbia Ministry of Health, 2016). The PharmaNet dataset captures all prescription medications dispensed by community pharmacies in BC and by hospital outpatient pharmacies for patients to use at home. Underlying causes of death were derived from Vital Statistics data using ICD-10 codes (Mattson et al., 2021, British Columbia Vital Statistics Agency, 2016).

### 2.2 Study design

Our objective was to establish a cohort of PWH who were dispensed OAT at least once (analytic cohort). First, a cohort of PWH aged 19 years or older with known gender was defined by considering all available provincial data in STOP HIV/AIDS between 2008 and 2020. The baseline date was adjusted to the later of two dates: 01 January 2008 or the earliest HIV-related record. Second, we followed individuals for a minimum of one year to identify any instances of OAT dispensations between 2008 and 2020 (Appendix 1*)*. The OAT dispensations were captured, according to the pertinent DINPIN, using the PharmaNet database (Appendix 2). OAT dispensations intended for pain management purposes were excluded, according to the corresponding transaction field in the PharmaNet database. The index date was assigned as the date of the first OAT dispensation during the study period; we excluded cases where a dispensation was initiated before the study period and either continued or ended during the study (a total of 761 episodes out of 14,413 episodes). In this context, the term “first OAT” signifies the first instance of OAT medication being prescribed and dispensed to individuals during the study time-frame. The endpoint for each individual observation period was determined by the earliest occurrence of one of the following: i) date of death; ii) date of last healthcare encounter based on administrative criteria of loss to follow-up (i.e., absence of any record in DTP, MSP, DAD, or PharmaNet databases for a minimum of 18 months); iii) March 31, 2020, which marked the end of the follow-up.

### 2.3 Assessment of OAT exposure

Treatment episodes were constructed for each OAT product (methadone [2008–2020], buprenorphine/naloxone [2008–2020], SROM [2015–2020], iOAT [2015–2020]). We used information from two fields in the PharmaNet database: service date (i.e., date of OAT dispensation) and days supplied (i.e., intended number of days that a dispensed product will last) (Pearce et al., 2020, British Columbia Ministry of Health, 2016). Continuous OAT episodes were defined as having no interruptions in prescribed doses lasting ≥ 2 days for SROM, ≥ 3 methadone and iOAT (i.e., diacetylmorphine and hydromorphone), or ≥ 6 days for buprenorphine/naloxone (College, 2022, British Columbia Centre on Substance Use and British Columbia Ministry of Health, 2017, Centre, 2020). To account for unidentified gaps observed in the PharmaNet data (e.g., treatment episodes occurring in hospitals or incarceration centers) certain assumptions were made. In cases where individuals were reinitiated on OAT at the same or higher dose after an observed gap, it was assumed that the treatment was continued during the gap period if the first dose after the gap was equal to or above the therapeutic dose. Individuals may change their OAT medication type during a continuous episode; if an episode consisted of multiple OAT medications, the medication type that was continued until the end of the continuous treatment episode was considered as the primary OAT medication for that episode (Appendix 3).

### 2.4 OAT uptake and treatment outcome

Treatment uptake was assessed as the proportion of individuals who were dispensed OAT during the study. Treatment retention was defined as any episode lasting ≥ 12 months. A five-year look-back window (LBW) was used to evaluate OAT dispensation and retention prior to the start of the study period (Nanditha et al., 2022). Therapeutic dose – an optimal dosing level that effectively prevents craving and withdrawal symptoms – was set at ≥ 60 mg for methadone, ≥ 240 mg morphine equivalent (MME) for SROM, ≥ 12 mg for Buprenorphine/Naloxone, ≥ 200 mg for iOAT (College, 2022, British Columbia Centre on Substance Use and British Columbia Ministry of Health, 2017, Centre, 2020).

### 2.5 Assessment of covariates

The final multivariable model considered several covariates. Gender (men, women), previous history of OAT retention (retained, not retained, no previous OAT), and lifetime diagnosis of chronic hepatitis C virus (HCV) from DTP (yes, no) were included as time-fixed categorical variables at the first OAT episode during the study period. Psychotic disorders (yes, no), diabetes mellitus (yes, no), and HIV viral load suppression (yes, no, unknown) were measured as categorical time-varying variables at the beginning of each new OAT episode. Psychotic disorders were determined based on one applicable hospitalization (DAD) or two healthcare encounters (MSP) within a twelve-month period using specific diagnostic codes, as previously described (Yazdani et al., 2022, Chronic Disease Information Working Group, 2018). Diabetes mellitus was identified based on various criteria, including hospitalizations (DAD), healthcare encounters (MSP), insulin, and oral antihyperglycemic prescriptions (PharmaNet), all within a twelve-month period (Chronic Disease Information Working Group, 2018). The HIV viral load was defined as “suppressed” if the values for two consecutive tests, taken 90 days apart, were < 200 copies/ml. OAT medication type (methadone, buprenorphine/Naloxone, SROM, iOAT) was assessed at the end of the OAT episode. Therapeutic dose achievement (yes, no) was determined based on specific dose thresholds for each medication type. Median values, 25th percentile (Q1), and 75th percentile (Q3) were reported for continuous variables, including age at first OAT episode and total days on OAT during the study. A list of all variables examined in this study can be found in Appendix 4, providing a detailed description of each variable.

### 2.6 Statistical analysis

The study conducted a comparison of characteristics among PWH who were initiated on different OAT medications. To account for the availability of different OAT medications during different time periods, the sample characteristics were assessed based on the first OAT episode in two distinct eras: 2008–2014 (Appendix 5) and 2015–2020. The Wilcoxon-Mann Whitney test was used for continuous variables, while the chi-square or Fisher's exact test was used for categorical variables. The study examined the overall percentage of OAT uptake and specific medication types across calendar years. The retention rate was calculated by dividing the number of retained episodes by the total number of episodes. The study also assessed the time to achieve therapeutic dose for each OAT medication type at the first episode and across all treatment episodes over the calendar years. An unadjusted linear regression model was used to analyze the temporal trend in the retention rate over the years, overall, and for each OAT medication type.

Factors associated with OAT retention were examined using both univariable and multivariable logistic regression. To account for repeated measurements over time, a generalized estimating equation (GEE) approach was used. An interaction term between OAT medication type and HCV was included in the model. This interaction term aimed to evaluate whether the relationship between OAT medication type and retention odds is influenced by a lifetime diagnosis of HCV. To handle missing HIV viral load values, a multiple imputation technique was used, utilizing data from the DTP registry one year prior to OAT dispensation, with 291 missing values imputed out of 720. In the multivariable model, covariates were first included if they showed significant differences between the retained and non-retained episodes in the univariate model. The final selection of covariates was performed using a backward elimination process based on the Akaike Information Criterion (AIC) and Type III p-values (Lima et al., 2010).

All statistical analyses were performed using SAS version 9.4 (SAS, Cary, NC, USA).

## 3 Results

Between January 2008 and March 2020, 10,959 individuals met the eligibility criteria for HIV. Of these, 13.8% (n = 1,515) of individuals received at least one OAT dispensation (analytic cohort). These data reflect that < 20% of PWH in BC in 2008–2020. Table 1 describes the comparison of covariates among individuals who had their initial episodes of methadone, buprenorphine/naloxone, SROM, and iOAT between 2015 and 2020. There were statistically significant differences across both gender (p < 0.01) and age (p < 0.0001) for different OAT medications. The majority of individuals who started buprenorphine/naloxone, SROM, and iOAT were men, accounting for over two-thirds of the respective groups. In contrast, there was a more balanced gender distribution among individuals who started on methadone, with 57.6% (n = 417) being men and 42.4% (n = 307) being women. The prevalence of comorbidities except for mood and anxiety (p = 0.90) disorder and osteoarthritis (p = 0.21) varied significantly, indicating that individuals on different types of OAT medication had different patterns of comorbid conditions. For instance, over 50% of individuals receiving methadone, buprenorphine/naloxone, and SROM had a lifetime diagnosis of HCV. Whereas, the prevalence of HCV among those receiving iOAT was relatively lower, at 34%. Appendix 5 provides a comparison of covariates among individuals who initiated methadone and buprenorphine/naloxone episodes between 2008 and 2014.

**Table 1:** The sample characteristics of PWH with OAT episodes in 2015–2020, categorized by first OAT Prescription type.

	**Overall** **(n = 1,096)**	First OAT Prescription Type*in 2015–**2020**				
**Sample Characteristics**		**Methadone** **(n = 724)**	**Buprenorphine** **(n = 262)**	**SROM** **(n = 66)**	**iOAT** **(n = 44)**	p-value**
**Gender**^a^ Men Women	677 (61.7)419 (38.2)	417 (57.6)307 (42.4)	183 (69.8)79 (30.1)	43 (65.1)23 (34.8)	34 (77.2)10 (22.7)	**<0.01**
**Age,** years,median (Q1, Q3)	45 (38, 52)	44 (38, 51)	44 (36,52)	50 (43, 57)	53 (46, 60)	**<0.0001**
**Ever retained (in 12 months)**^b^ No Yes No previous OAT Rx)	209 (19.0)383 (34.9)504 (45.9)	149 (20.5)301 (41.5)274 (37.8)	42 (16.0)54 (20.6)166 (63.3)	16 (24.2)20 (30.3)30 (45.4)	<5 c 5–10 (15–20) c34 (77.2)	**<0.0001**
**Comorbidities (not mutually exclusive)**^d^						
						
Psychotic Disorders	169 (15.4)	95 (13.1)	56 (21.3)	15 (22.7)	<5	**<0.01**
Mood & Anxiety Disorders	130 (11.8)	90 (12.4)	29 (11.0)	7 (10.6)	<5	0.90
Chronic Pain Conditions	510 (46.5)	316 (43.6)	127 (48.4)	37 (56.0)	30 (68.1)	**<0.01**
Hepatitis C Virus	596 (54.3)	379 (52.3)	160 (61.0)	42 (63.6)	15 (34.0)	**<0.01**
Cardiovascular Diseases	51 (4.6)	28 (3.8)	13 (4.9)	<5	7 (15.9)	**0.01**
COPD	49 (4.4)	24 (3.3)	20 (7.6)	<5	<5	**0.02**
Diabetes Mellitus	49 (4.4)	22 (3.0)	16 (6.1)	<5	9 (20.4)	**<0.0001**
Cancer	43 (3.9)	15 (2.0)	16 (6.1)	<5	8 (18.1)	**<0.0001**
Osteoarthritis	53 (4.8)	30 (4.1)	14 (5.3)	5 (7.5)	<5	0.21
Liver Diseases	242 (22.0)	143 (19.7)	72 (27.4)	19 (28.7)	8 (18.1)	**0.03**
**Suppressed Viral Load** (<200 mg/copies) e No Yes Unknown	184 (16.7)508 (46.3)404 (36.8)	118 (16.3)324 (44.7)282 (38.9)	47 (17.9)135 (51.5)80 (30.5)	16 (24.2)22 (33.3)28 (42.4)	<527 (61.3) 10–15 (30–35)	**0.02**
**Prescriber Type**^f^ General Practitioner Specialist Physicians Community Medicine Others Unknown	899 (82.0)42 (3.8)88 (8.0)11 (1.0)56 (5.1)	614 (84.8)21 (2.9)47 (6.4)7 (0.9)35 (4.8)	206 (78.6)20 (7.6)27 (10.3) <5 c 5–10 (1–5) c	53 (80.3) <5 <56 (9.0)6 (9.0)	26 (59.0) <58 (18.1) <57 (15.9)	**<0.0001**
**Starting Dose**, g/ml, median (Q1, Q3)		35.8 (30, 50)	10 (5, 10)	200 (60, 200)	145 (100, 333.3)	
**Time to therapeutic dose, days** (if achieved, n = 364) g	15 (6, 34.5)	15 (7, 30)	15 (3, 45)	15 (5, 40)	5 (1,10)	0.44

All variables are measured at first OAT episodes during the study timeframe. The term “first OAT episode” refers to the treatment episode constructed according to the initial dispensing of OAT medication following the individual's entry into the HIV cohort. Any OAT episodes that were initiated before the entry into the HIV cohort and either continued or ended during the study period were excluded from the analysis. Unless stated otherwise, the values are expressed as n (%).

Abbreviations: OAT: opioid agonist therapy; SROM: slow-release oral morphine; iOAT: injectable opioid agonist therapy; PWH: people with HIV; Q1: 25% interquartile; Q3: 75% interquartile; COPD: chronic obstructive pulmonary disease; DINPIN; Drug/Product Identification Number; ICD-9/9-CM/10-CA: International Classification Disease 9th Revision /9th Revision, Clinical Modification/ 10th Revision, Canada; MSP: Medical Services Plan; DAD: Discharge Abstract Database; DTP: Drug Treatment Program; MME: morphine milligram equivalent.

* The first OAT prescription type is according to the first OAT Rx in the first treatment episode.

** The p-value shows the comparison of variables among individuals with their first episodes of methadone, buprenorphine/naloxone, SROM, and iOAT in 2015–2020.

a The variable “gender” encompasses both cisgender and transgender individuals who identify as men or women.

b Ever history of retention was assessed using a five-year lookback window. For each treatment episode, retention was assessed based on treatment duration, defined as no interruption in the prescribed doses for at least 12 months.

c Values have been censored and masked for privacy reasons.

d Comorbid conditions were defined using pertinent diagnostic codes (ICD- 9/9-CM/10-CA codes or BC-specific codes/non-ICD diagnostic doses) in MSP, DAD, or DINPINs in PharmaNet data. A detailed description of covariates appears in Appendix 4.

e The HIV viral load suppression was defined as suppressed if the values for two consecutive tests, 90 days apart were < 200 copies /ml using the HIV DTP registry.

f The variable prescriber type was defined based on the “provider specialty” field in MSP.

g Measured across all “first” OAT episodes during the study (i.e., ≥ 60 mg for methadone, ≥ 12 mg for Buprenorphine/Naloxone, 240 MME for SROM, 200 mg for iOAT).

Out of 1,096 treatment episodes initiated in 2015–2020, 33.2% (n = 364) reached therapeutic dose. The median time to therapeutic dose did not differ significantly between OAT medication types in the first treatment episode (p = 0.44) (Table 1). Across all treatment episodes during the study, the median time to therapeutic dose was 14 days (Q1-Q3: 7–30) for methadone, 10 days (Q1-Q3: 4–29) for buprenorphine/naloxone, 2 days (Q1-Q3: 1–5) for iOAT, and 10 (Q1-Q3: 3–21) days for SROM.

**Fig. 1:**
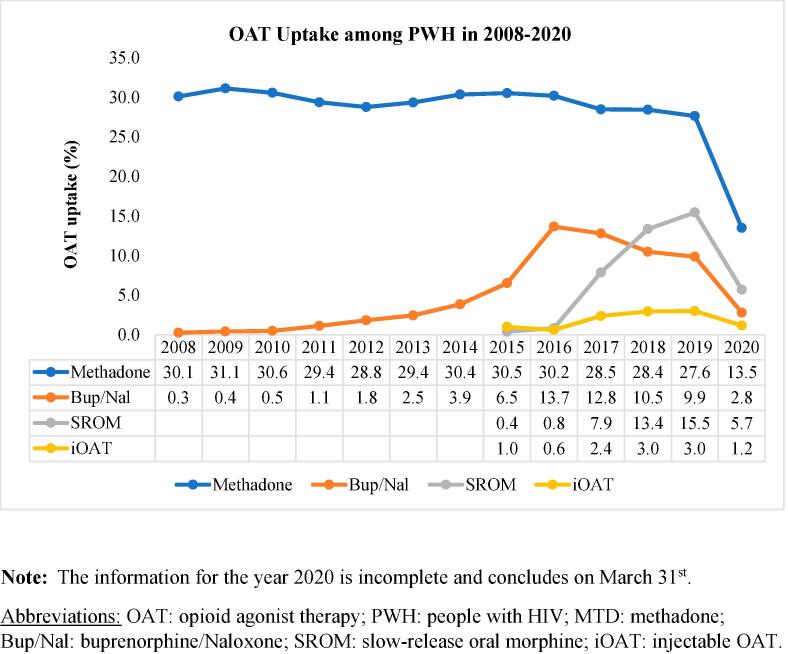
The uptake of OAT among PWH in 2008–2020, categorized by OAT medication type. Note: The information for the year 2020 is incomplete and concludes on March 31st., Abbreviations: OAT: opioid agonist therapy; PWH: people with HIV; MTD: methadone; Bup/Nal: buprenorphine/Naloxone; SROM: slow-release oral morphine; iOAT: injectable OAT.

In 2008, 30.2% (n = 338) of PWH received OAT. The uptake increased to a peak of 35.5% (n = 458) in 2015 and further rose from 39.2% (n = 508) to 45.9% (n = 535) between 2016 and 2019. The uptake of buprenorphine/naloxone, SROM, and iOAT experienced an increase over the calendar years. Buprenorphine/naloxone uptake reached its highest point in 2016 at 13.7% (n = 177), after which it gradually declined in subsequent years. In contrast, the uptake of methadone remained relatively consistent (Fig. 1*,* Appendix 6).

**Fig. 2:**
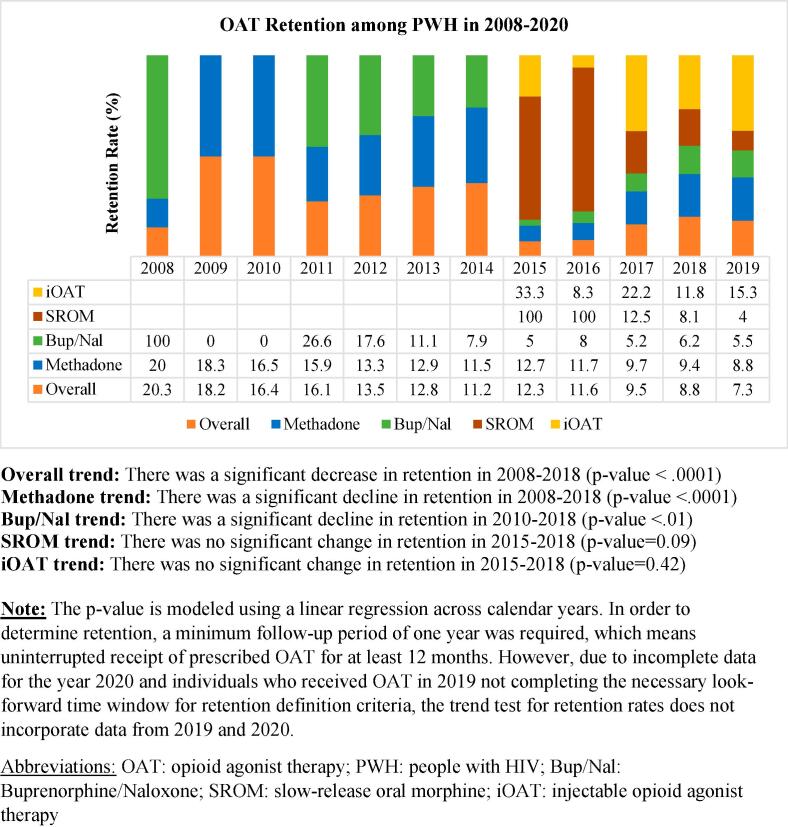
OAT retention rates among PWH in 2008–2020, categorized by OAT medication type, Overall trend: There was a significant decrease in retention in 2008–2018 (p-value < 0.0001), Methadone trend: There was a significant decline in retention in 2008–2018 (p-value < 0.0001), Bup/Nal trend: There was a significant decline in retention in 2010–2018 (p-value < 0.01), SROM trend: There was no significant change in retention in 2015–2018 (p-value = 0.09), iOAT trend: There was no significant change in retention in 2015–2018 (p-value = 0.42), Note: The p-value is modeled using a linear regression across calendar years. In order to determine retention, a minimum follow-up period of one year was required, which means uninterrupted receipt of prescribed OAT for at least 12 months. However, due to incomplete data for the year 2020 and individuals who received OAT in 2019 not completing the necessary look-forward time window for retention definition criteria, the trend test for retention rates does not incorporate data from 2019 and 2020., Abbreviations: OAT: opioid agonist therapy; PWH: people with HIV; Bup/Nal: Buprenorphine/Naloxone; SROM: slow-release oral morphine; iOAT: injectable opioid agonist therapy.

We identified 11,888 OAT episodes in 2008–2020, with a retention rate of 12.8% (n = 1,522). The overall retention for OAT decreased by 56.6% from 2008 (20.3%) to 2018 (8.8%). The retention rate for methadone showed a significant decline over the reported years, ranging from 20% in 2008 to 9.4% in 2018 (p-value < 0.0001). Similarly, the retention of buprenorphine/naloxone also experienced a statistically significant decrease from 2011 (26.6%) to 2018 (6.2%) (p < 0.01). In 2015 and 2016, individuals receiving SROM had a retention rate of 100%. However, the retention rate declined from 12.5% in 2017 to 8.1% in 2018. Although the trend test did not reach statistical significance (p = 0.09), there was a noticeable decrease in the SROM retention rate in 2015–2018. In contrast, the retention rates for individuals receiving iOAT did not show significant changes in 2015–2018 (p = 0.42) (Fig. 2*,* Appendix 7).

The results of the multivariable model are presented in Table 2. The findings are presented as odds ratios (OR) along with a 95% confidence interval (CI). Buprenorphine treatment had significantly lower odds of retention (OR: 0.58; 95% CI: 0.36–0.92) compared to methadone. In contrast, no change in odds of retention was observed for SROM (0.72; 0.33–1.54) and iOAT (0.81; 0.31–2.12). Interestingly, the effect of OAT medication type on retention odds was found to be modified by HCV. Individuals who received iOAT and had HCV were 3.6 times more likely to be retained (3.61; 1.20–10.83), compared to those receiving methadone with no HCV. On the other hand, individuals receiving SROM who had HCV were 42% less likely (0.58; 0.34–0.98) to retain. Additional factors found to be associated with increased odds of retention include: a 10-year increase in age (1.69; 1.46–1.95), previous history of retention (1.96; 1.40–2.73), imputed suppressed HIV viral load (1.35; 1.10–1.67), increased number of days on OAT during the study (1.04; 1.00–1.07). Achieving minimum therapeutic dose was associated with 8.2 fold increase in retention odds (8.22; 6.67–10.14). The univariate model is presented in Appendix 8.

**Table 2:** Factors associated with OAT retention among PWH in British Columbia.

	**Adjusted Model** **OR (95% CI)**
Interaction Term: OAT Type * HCV	
Methadone and no HCV [ref] Methadone and HCV Bup/Nal and no HCV Bup/Nal and HCV SROM and no HCV SROM and HCV iOAT and no HCV iOAT and HCV	1.000.96 (0.74–1.24) **0.58 (0.36**–**0.92)**0.78 (0.45–1.35)0.72 (0.33–1.54) **0.58 (0.34**–**0.98)**0.81 (0.31–2.12) **3.61 (1.20**–**10.83)**
**Time-Fixed Variables**^a^	
**Age** (per 10-year increase)	**1.69 (1.46**–**1.95)**
**HCV (lifetime)** No [ref] Yes	Interaction Term OAT type *HCV
**Ever Retained, using a 5-year LBW** No Yes No OAT Before Entering	1.00 **1.96 (1.40**–**2.73)** **1.69 (1.22**–**2.34)**
**Time-varying Categorical Variables**^b^	
Total days on OAT during the study (per 365.25 days increase)	**1.04 (1.00**–**1.07)**
OAT Type ^b*^ Methadone [ref] Bup/Nal SROM iOAT	Interaction Term OAT type *HCV
**Diabetes Mellitus** No [ref] Yes	1.000.60 (0.32–1.13)
**Imputed HIV Viral Load suppression (copies/ml)** Not Suppressed [ref] Suppressed < 200 Unknown c	1.00 **1.35 (1.10**–**1.67)**1.34 (0.94–1.92)
**Therapeutic Dose**^d^	
No [ref] Yes	1.00 **8.32 (6.67**–**10.14)**

**Note:** To model retention, Logistic regression with Generalized Estimating Equations (GEE) is utilized, taking into consideration repeated measurements. This approach aims to estimate the probability that “retained” equals 1; If in the univariate model, the covariates did not differ significantly between retained and non-retained episodes, they were not used for selection by the model (Appendix). This model is built using all episodes; covariates were selected based on Type III p-values and Quasi-Akaike Information Criterion (QIC). A detailed description of covariates is provided in Appendix 4.

Abbreviations: OAT: opioid agonist therapy; PWH: people with HIV; Bup/Nal: Buprenorphine/Naloxone; OR: odds ratio; CI: confidence interval; Q1: 25% interquartile; Q3: 75% interquartile; HCV: hepatitis C virus; LBW: look-back window.

a Time-fixed variables measured at first OAT during the study period.

b Time-varying variables are measured at the beginning of each new OAT episode except for OAT type ^b*^ which was measured as of the end of each OAT episode.

c Missing viral load values (291 out of 720) were imputed using the most recent data available within a year before OAT dispensation from the DTP registry.

d Assessed across all treatment episodes (i.e., ≥ 60 mg for methadone, ≥ 12 mg for Buprenorphine/Naloxone, ≥ 240 MME for SROM, 200 mg for iOAT). We lacked information on the drug strength for “66123367,” which accounted for 25% of iOAT episodes. However, this did not affect retention evaluation. We assumed a therapeutic dose based on other DINPINs. There were 0.71% of episodes with unknown status, imputed as “yes” in the modeling stage.

## 4 Discussion

An increase in overall OAT uptake and the number of treatment episodes across calendar years was observed. Specifically, there was a notable rise in iOAT and SROM uptake in more recent years. Albeit, there was a statistically significant decline in overall retention, and methadone and buprenorphine/naloxone retention. However, the retention rates for iOAT and SROM did not exhibit significant changes over the calendar years.

Compared to methadone, the odds of retention for buprenorphine/naloxone were significantly low, whereas no change in the odds of retention was observed for SROM and iOAT. Our data corroborate with recent evidence in Canada (Kurz et al., 2022). Krebs et al., found consistently higher rates of buprenorphine/naloxone discontinuation in BC (Krebs et al., 2021). Another study in Nova Scotia found significantly higher dropouts and lower retention for treatment with buprenorphine/naloxone (Sadek and Saunders, 2022).

We found that the odds of retention in various OAT types were influenced by a lifetime HCV diagnosis. PWH who had HCV showed substantially higher odds of retention when receiving iOAT compared to PWH with no HCV who were receiving methadone. The impact of HCV on OAT retention is a multifaceted aspect that falls outside the scope of this study. Nevertheless, our data suggest that individuals with concurrent HCV infection exhibit improved participation in iOAT. Additional factors found to be associated with increased odds of 12-month retention include: a 10-year increase in age, achieving a therapeutic dose, previous history of OAT retention, suppressed HIV viral loads, and longer duration of OAT participation during the study period.

The increase in OAT uptake and treatment episodes aligns with the previous research reporting temporal improvement in OUD cascade of care in BC (Socías et al., 2020). Higher OAT uptake in the recent calendar years reflects efforts aimed at expansion of access to low-threshold and supervised programs as part of the BC province response to the overdose crisis, particularly during the period of substantially increased fentanyl use (Officer, 2022). The rise in the utilization of SROM and iOAT from 2017 onwards further supports the possibility of treatment re-attempts and a rise in the prevalence of severe OUD cases (Tahsin et al., 2022).

Despite a notable increase in OAT uptake, aligned with previous studies, no improvement in retention outcomes was observed over time (Tahsin et al., 2022, Socías et al., 2018). In fact, our findings suggest notably low retention among PWH, dropping to < 10% during the period of increased fentanyl consumption. This low retention could be attributed to regulatory changes in BC OAT program in February 2014 (British Columbia Coroners Service, 2019), increased tolerance, and contaminated illicit drug supply (Socías et al., 2020, Socías et al., 2018).

Our study extends the previous literature, by identifying additional factors associated with retention in OAT, especially among PWH. With an almost 8-fold increase in odds of retention, our data highlight the role of optimal OAT dosing in improving retention outcomes. Specifically, our findings suggest that supporting clients to reach dosage thresholds of at least 60 mg/day for methadone, 12 mg/day for buprenorphine/naloxone, 240 MME for SROM, and 200 mg/day for iOAT can significantly improve retention rates. Our data indicate that the median time to therapeutic dose did not differ across calendar years for different types of OAT. However, iOAT demonstrated the shortest time to therapeutic dose compared to other OAT options. This suggests that iOAT may offer a faster route to achieving the optimal dosage for effective treatment.

We acknowledge limitations of the present study necessitating cautious interpretation of the results. First, our findings failed to indicate a faster induction schedule for buprenorphine compared to methadone The PharmaNet data do not capture in-hospital OAT dispensations. Therefore, overestimation of time to therapeutic dose is possible if the therapeutic dose was achieved during a hospitalization episode. Continuation of OAT treatment in hospital is particularly more plausible for PWH initiating buprenorphine/naloxone, given the higher burden of comorbid conditions at OAT initiation (Table 1*,* Appendix 5). Additionally, time to therapeutic dose was assessed among individuals who did not already initiate their treatment episodes at the set therapeutic dose. It is worth noting that optimal OAT dosing heavily relies on individual differences such as metabolism, comorbidities, and drug-drug interactions, and therefore, should be based on clinical judgment. Nonetheless, the OAT therapeutic dose was defined according to the OUD prescribing guidelines in BC (British Columbia Centre on Substance Use and British Columbia Ministry of Health, 2017). Second, due to the inherent nature of administrative data, we were unable to measure the effects of individual socio-economic variables such as employment and ethnicity previously shown to be key predictors of sub-optimal retention (Socías et al., 2020). However, for the data that were available, we carefully examined covariates known to be associated with retention and imputed variables with incomplete measures. Third, we should be cautious about the generalizability of our findings to individuals receiving OAT without HIV or receiving OAT in other settings, especially in jurisdictions with a different healthcare system.

This study's strength is its population-based approach, which thoroughly examines the characteristics of OAT treatment among PWH. It takes place in a universal healthcare system where PWH receive free HIV care and OAT programs. The consistency of our findings with the recent evidence emerging in Canadian settings is reassuring.

## 5 Conclusion

In a universal healthcare setting, we observed low OAT retention rates among PWH. There was a significant decline in overall OAT, methadone, and buprenorphine/naloxone retention. However, retention outcomes were more favorable for PWH receiving SROM and iOAT. It is important to consider comorbidities when initiating OAT in PWH. Our data showed higher odds of retention for PWH with HCV receiving iOAT, while lower odds were observed for those receiving SROM. Increasing age, previous retention history, suppressed HIV viral load and achieving the therapeutic dose were associated with higher odds of retention. These findings underscore the need for optimal management of OUD among PWH, using integrated models of care that maximize retention and achieve therapeutic doses. Future studies must aim at continued evaluation of retention outcomes post 2020, which presents the intersectionality of overdose crisis aggravation, regulatory changes in BC OAT programs, and the advent of COVID-19 pandemic.

## Author contributions

KY and KS contributed to the research conceptualization. KY and KS were responsible for the development and design of methodology, and data interpretation. KY was responsible for original draft preparation, visualization, data presentation, and project coordination. MY, KD, and JT were responsible for data curation, data cleaning, statistical analysis, programming, implementation of computer code, and statistical interpretation. JM, and RB, were responsible for the acquisition of financial support and the provision of research resources. RJ, SE, JM, and RB contributed to data interpretation and critical review of the paper. All authors contributed to writing, editing, and manuscript development.

## Declaration of Competing Interest

The authors declare the following financial interests/personal relationships which may be considered as potential competing interests: Dr. Julio S. G. Montaner is the Executive Director and Physician-in-Chief of the BC Centre for Excellence in HIV/AIDS, a provincial program serving all BC health authorities, and based at St. Paul’s Hospital-Providence Health Care. JM’s Treatment as Prevention® (TasP®) research, paid to his institution, has received support from the BC Ministry of Health, Health Canada, Public Health Agency of Canada, Genome BC, Vancouver Coastal Health and VGH Foundation. Institutional grants have been provided by Gilead, Merck and ViiV Healthcare.
