# Supplementary material for: Characterizing opioid agonist therapy uptake and factors associated with treatment retention among people with HIV in British Columbia, Canada
Source: Prev Med Rep. 2023 Jun 29;35:102305. doi: 10.1016/j.pmedr.2023.102305 (PMC10382920; doi:10.1016/j.pmedr.2023.102305)
Supplement: Supplementary data 1 [file mmc1.docx]

Table of Contents

[**Appendix 1. Cohort Description** 2](#_Toc136869770)

[**Appendix 2. OAT Regulations in BC and Drug/Product Identification Number** 3](#_Toc136869771)

[**Appendix 3. OAT Episode Construction and Data Cleaning** 6](#_Toc136869772)

[**Appendix 4. Assessment of Covariates** 10](#_Toc136869773)

[**Appendix 5. Sample of PWH with OAT episodes initiation in 2008-2014** 14](#_Toc136869774)

[**Appendix 6. OAT Uptake, categorized by OAT medication type** 17](#_Toc136869775)

[**Appendix 7. OAT Characteristics, by OAT medication type among PWH in 2008-2020** 18](#_Toc136869776)

[**Appendix 8. Univariate Model** 22](#_Toc136869777)

# **Appendix 1. Cohort Description**

**Supplementary Table 1. Cohort description of PWH with at least one OAT dispensation in BC, using STOP cohort**

| **Cohort Description** | |
| --- | --- |
| All PWH in STOP Cohort in BC | 15,957 |
| All PWH residing in BC between 01 January 2008 – 31 March 2020 | 12,293 |
| PWH who are ≥19 years of age | 11,964 |
| Excluding PWH with unknown gender | 11,964 |
| PWH with minimum 12 months follow up | 10,959 |
| PWH with at least one OAT prescription during the follow-up | 1,515 |

***Study Baseline:** For individuals who joined the STOP HIV/AIDS cohort before 01 January 2008, their baseline date in the study was modified to January 1, 2008. In simpler terms, the later date between 01 January 2008, and the date of their earliest HIV-related record was chosen as the starting point for these individuals in the cohort. This adjustment guarantees uniformity in the baseline timeframe for all participants in the study, regardless of their entry into the STOP cohort before 2008.

****≥ 12 months follow-up:** By mandating a minimum of one year of follow-up, the study aims to capture sufficient data to analyze the occurrence and impact of OAT dispensation within the HIV cohort. The average duration from entry into the HIV cohort to the initiation of OAT during the study period was 26 months (Q1-Q3: 6-65). This finding provides further support for our requirement of a minimum follow-up period of more than 12 months.

Abbreviation: STOP: Seek and Treat for Optimal Prevention of HIV/AIDS; PWH: people with HIV; BC: British Columbia; OAT: opioid agonist therapy; Q1: 25% interquartile; Q3: 75% interquartile.

# **Appendix 2. OAT Regulations in BC and Drug/Product Identification Number**

**OAT Availability and Regulations**

The opioid agonist therapy (OAT) program in BC was established in 1996 and has undergone significant development and expansion over the years. Prior to 2008, oral solution methadone was the main pharmacotherapy for opioid use disorder (OUD) prescribed by licensed physicians. In 2008, Buprenorphine/Naloxone was added to the provincial drug formulary and recommended as the first-line treatment in 2017 due to its safety profile, lower risk of fatal overdose, and faster induction schedule. The slow-release oral morphine (SROM) was approved for use in Canada in November 2014 and recommended as an option for patients who have not responded to previous treatments. Injectable OAT (iOAT) with diacetylmorphine (pharmaceutical-grade heroin) was available in research settings before 2016. It was formally approved in BC under supervised conditions in May 2016, and hydromorphone was formally approved by the government of Canada in May 2019 as a second injectable OAT medication to treat severe OUD.

**Supplementary Table 2. Product Identification Numbers (PINs) and Drug Identification Numbers (DINs) for PharmaCare Claims for OAT Treatment**

| **OAT Medication Type** | **DINPIN ^1,2^** |
| --- | --- |
| **Methadone** | |
| Methadose 10mg/mL (cherry) | 66999997 |
| Methadose 10mg/mL (cherry) | 66999999 |
| Methadose 10mg/mL (cherry) | 67000000 |
| Methadose 10mg/mL (cherry) | 66999998 |
| Methadose10mg/mL Sugar- Free | 67000001 |
| Methadose10mg/mL Sugar-Free | 67000003 |
| Methadose10mg/mL Sugar- Free | 67000002 |
| Methadose10mg/mL Sugar-Free | 67000004 |
| Methadone 10mg/ml (Sterinova®) | 67000017 |
| Methadone 10mg/ml (Sterinova®) | 67000018 |
| Methadone 10mg/ml (Sterinova®) | 67000019 |
| Methadone 10mg/ml (Sterinova®) | 67000020 |
| Compounded methadone 10 mg/mL  with direct interaction | 67000013 |
| Compounded methadone 10 mg/mL  with direct interaction | 67000014 |
| Compounded methadone 10 mg/mL  without direct interaction | 67000015 |
| Compounded methadone 10 mg/mL  without direct interaction | 67000016 |
| Other Codes for Methadone | 999792; 999793; 66999990;6699999266999993;66999991; 67000005;67000007  67000006; 67000008 |
| **Buprenorphine/Naloxone (Suboxone)** | |
| Buprenorphine/Naloxone SL 2mg/0.5mg generics | 2453908 |
| Buprenorphine/Naloxone SL 2mg/0.5mg generics | 2424851 |
| Buprenorphine/Naloxone SL 8mg/2mg generics | 2453916 |
| Buprenorphine/Naloxone SL 8mg/2mg generics | 2424878 |
| Suboxone   Buprenorphine/Naloxone  2mg/0.5mg | 2295695 |
| Suboxone   Buprenorphine/Naloxone SL  8mg/2mg | 2295709 |
| Suboxone   Buprenorphine/Naloxone SL12mg/3mg | 2468085 |
| Suboxone   Buprenorphine/Naloxone SL 16mg/4mg | 2468093 |
| Other Codes for Buprenorphine/Naloxone | 2408090; 2408104;  242964; 2242963;  2242964; 66999994;  66999995; 66999996; 2502313; 2502348; 2502356 |
| **Slow Release Oral Morphine (Kadian)** | |
| Kadian 10 mg Capsule | 22123349 |
| Kadian 100 mg Capsule | 22123348 |
| Kadian 20 mg Capsule | 22123346 |
| Kadian 50 mg Capsule | 22123347 |
| **Injectable OAT** | |
| Diacetylmorphine 100 mg/ml | 22123357 |
| Hydromorphone 50 mg/ml | 2146126 |
| Hydromorphone 50 mg/ml | 2469413 |
| Narcotic Compound (PharmaCare non-benefit) | 66123367 |

**Note:** We encountered a lack of information regarding the drug strength for the specific identifier "66123367," which accounted for approximately 25% of all episodes related to iOAT. However, this particular issue did not pose a challenge when evaluating participant retention. In order to estimate therapeutic dosages, we made an assumption based on the achieved results from other DINPINs. Despite this, there were still 0.71% of episodes where the status remained unknown. To address this, during the multivariable modeling stage, we employed imputation techniques and assigned these unknown values as "yes."

**References:**

**1.** BC Pharma<https://www2.gov.bc.ca/gov/content/health/practitioner-professional-resources/pharmacare/pharmacies/product-identification-numbers/oat-pins-and-dins>

**2**. BC PharmaCare Formulary Research: https://pharmacareformularysearch.gov.bc.ca/

**3.** Kurz M, Min JE, Dale LM, Nosyk B. Assessing the determinants of completing OAT induction and long-term retention: A population-based study in British Columbia, Canada. J Subst Abuse Treat. 2022; 133:108647. doi:10.1016/j.jsat.2021.108647

# **Appendix 3. OAT Episode Construction and Data Cleaning**

**PharmaNet Data Cleaning**

To ensure error-free and accurate OAT records in the PharmaNet data, careful cleaning processes were implemented to identify possible errors and correct them. We primarily implemented a cleaning procedure described by Pearce L., and colleagues (*Supplementary* *Figures 1&2*). Additionally, we manually corrected inconsistent values within a consistent pattern of treatment. We identified 4,288,289 rows in the PharmaNet data and identified 180,330 errors of which 38,118 were corrected.

**References:**

**1.** British Columbia Centre on Substance Use and British Columbia Ministry of Health. A Guideline for the Clinical Management of Opioid Use Disorder (2017). Available at: "http://www.bccsu.ca/care-guidance-publications/"

**2.** Pearce LA, Min JE, Piske M, Zhou H, Homayra F, Slaunwhite A, Irvine M, McGowan G, Nosyk B. Opioid agonist treatment and risk of mortality during opioid overdose public health emergency: population based retrospective cohort study. Bmj. 2020 Mar 31; 368.


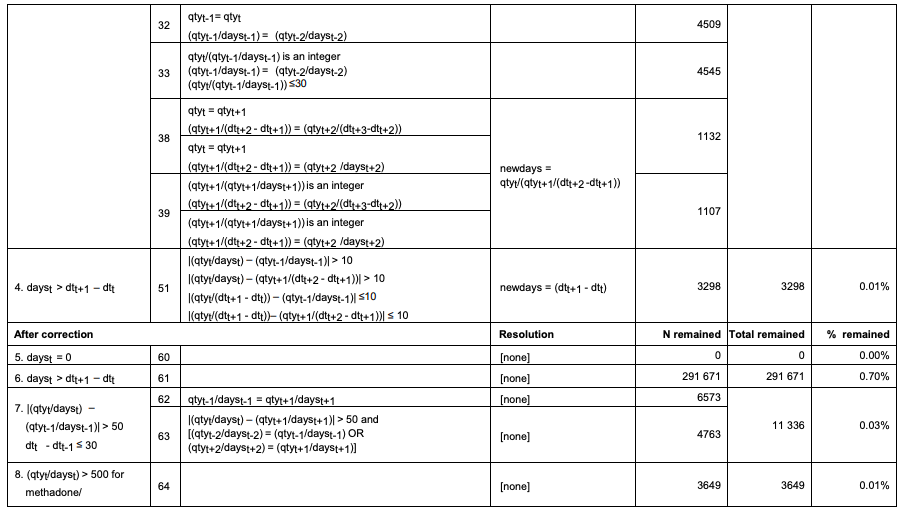
**Supplementary Figure 1. Cleaning procedure implemented by Pearce L., and colleagues (2020)**
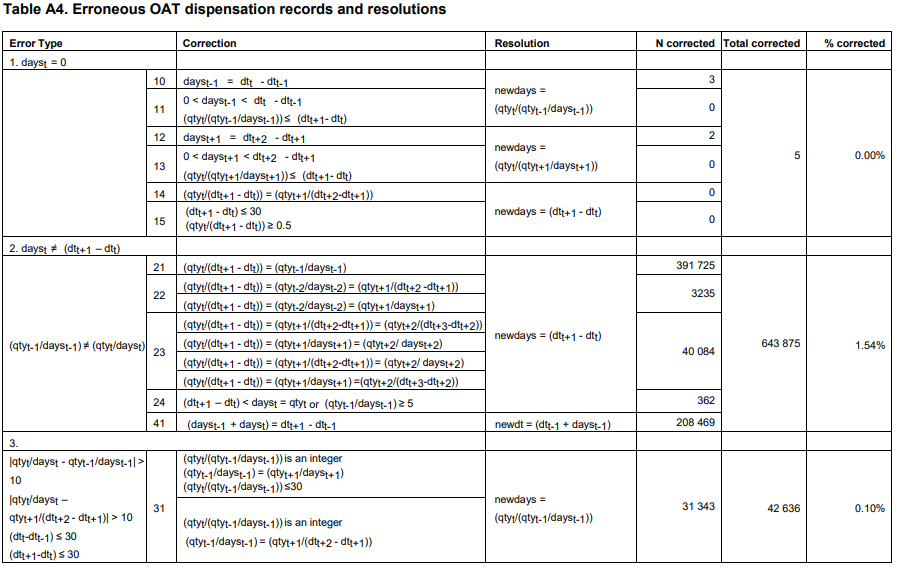


**Supplementary Figure 2. Diagram indicating OAT construction**

***Pearce et el., 2020***

**Footnote:** In our analysis, a continuous OAT episode was defined as having no interruptions in prescribed doses lasting ≥ 3 days for methadone and injectable OAT, ≥ 2 days for SROM, and lasting ≥ 6 days for buprenorphine/naloxone.

Abbreviations: MET: methadone; SROM: slow-release oral morphine; BNX: buprenorphine/naloxone; OAT: opioid agonist therapy

**Gap in treatment**

The PharmaNet database does not capture OAT dispensations occurring in hospitals, or incarceration centres. If OAT treatment is interrupted or discontinued, for safety reasons, individuals will have to restart the treatment at a lower dose than the previous stabilization dose. Therefore, in cases where individuals were reinitiated on OAT at the same or higher dose after an observed gap between treatment episodes, it was assumed that the treatment was continued during the gap period. This assumption was made if the first dose after the gap was a therapeutic dose or higher. However, if the first dose after the gap was below the therapeutic dose, it was counted as a new episode, indicating a restart of the treatment. Here are some instances where we have presented examples of this assumption, specifically focusing on methadone.

**MTD dx 1**

**40 mg**

**MTD dx 2**

**50 mg**

**MTD dx 3**

**40 mg**

2 days

5 days

ep. 1

ep. 2

**Case 1.** Gap in treatment & decrease in dose

**MTD dx 1**

**40 mg**

**MTD dx 2**

**50 mg**

**MTD dx 3**

**60 mg**

2 days

5 days

ep. 1

**Case 2.** Gap in treatment & increase in dose to therapeutic dose

**MTD dx 1**

**40 mg**

**MTD dx 2**

**50 mg**

**MTD dx 3**

**40 mg**

2 days

5 days

ep. 1

ep. 2

**Case 3.** Gap in treatment & increase in dose to below therapeutic dose

**Multiple OAT dispensations on the same day**

In some cases, individuals may have an indication of multiple OAT dispensations on the same day in the PharmaNet data. We were interested in time to therapeutic dose. Therefore, accurate calculation of the dispensed quantity per day (and not per Rx) was of value to our analysis in the cases of multiple dispensations on the same day.

We speculated that the same-day multiple dispensations occurred for the following reasons:

- *Split-dose*: an individual may take their OAT at different times during the day. If the OAT products and supply days dispensed on the same day were similar, we assumed cases of split-dose and added up doses to calculate the total dispensed quantity for that product per day.
- *Early refill*: an individual may have refilled their next prescription early. If supply days of OAT products dispensed on the same day were different, we assumed cases of early refill and did not add doses.
- *Human error*: wherever possible, we manually corrected for possible errors and if they were cases of split doses, we added up doses. Otherwise, no change in the doses was done.

# **Appendix 4. Assessment of Covariates**

**Supplementary Table 3. Covariates Assessment and Case-Finding Algorithms**

| **Comorbidities** | **Case-Definition** |
| --- | --- |
| Mood and Anxiety Disorder**^1^** | Episodic case-definition was used _One hospitalization OR two physician visits in one year, with ICD code(s) specified below, followed by further condition-related codes in subsequent years. The case definition applies to persons aged 1 and older.  **ICD-9/9-CM/10-CA Description**  **F30:** Manic episode  **F31:** Bipolar affective disorder  **F32:** Depressive episode  **F33:** Recurrent depressive disorder  **F34:** Persistent mood [affective] disorders  **F38**: Other moods [affective] disorders  **F39:** Unspecified mood [affective] disorder  **F40:** Phobic anxiety disorders  **F41**: Other anxiety disorders  **F42:** Obsessive-compulsive disorder  **F43:** Reaction to severe stress, and adjustment disorders  **F44:** Dissociative (conversion) disorders  **F45:** Somatoform disorders  **F48:** Other neurotic disorders  **F68:** Other disorders of adult personality & behavior  **296:** Affective psychoses  **300:** Neurotic disorders  **311:**Depressive disorder, not elsewhere classified  **50B (a BC-specific, non-ICD code):** Anxiety/Depression |
| Psychotic Disorders**^2^** | One hospitalization or two physician encounters in one year, with ICD code(s) specified below  **ICD-9/9-CM/10-CA Description**  **F25:** Schizoaffective disorders  **F20:** Schizophrenia  **F22:** Delusional Disorder  **F11.5-19.5,** **excl F17.5**): Psychotic Disorders Due to Psychoactive Substance Use  **F21:** Schizotypal Disorders  **F24:** Induced delusional disorder  **F23:** Acute and Transient Psychotic Disorders  **F28:** Other Nonorganic Psychotic Disorders  **F29:** Unspecified Nonorganic Psychosis  **292:** Drug-induced Psychosis  **292.1** Drug-induced Psychosis  **295:** Schizophrenic disorders  **297:** Paranoid States  **298:** Other Organic Psychoses |
| Chronic Pain**^3^** | Based on ICD codes in MSP (physician encounters) & DAD (hospitalizations); One instance of code was needed in the "highly likely" category, OR two in the "likely" separated by at least 30 days  **Highly Likely:** 338.2; 338.21; 338.22; 338.28; 338.29; 338.4  **Likely:** 307.8; 307.89; 338; 719.41; 719.49; 719.45; 719.46; 719.47; 720; 720.2; 720.9; 721; 721.1; 721.2; 721.3; 721.41; 721.42; 721.6; 721.8; 721.9; 721.91; 722; 722.1; 722.11; 722.2; 722.3; 722.31; 722.32; 722.39; 722.4; 722.51; 722.52; 722.6; 722.7; 722.71; 722.73; 722.8; 722.81; 722.82; 722.83; 722.9; 722.91; 722.92; 722.93; 723-723.9; 724-724.9; 724.01; 724.02; 724.09; 724.79; 729-729.2; 729.4; 729.5 |
| CVD**^4^** | Excel File _ CVD Sheet |
| COPD**^5^** | Excel File _ COPD Sheet |
| Diabetes  Mellitus**^6^** | Excel File _ DM Sheet |
| Osteoarthritis**^7^** | Excel File _ Cancer Sheet |
| Liver Disease**^8^** | Excel File _ Liver Diseases |
| Cancer**^9^** | BC Cancer Agency, identifying non-AIDS defining cancer as cancer cases other than Kaposi sarcoma, non-Hodgkin's lymphoma, and cervical cancer.” |
| HCV & suppressed viral load**^10^** | Based on available diagnoses from DTP in the STOP cohort |
| Prescriber Type | Based on “provider specialty” field in the MSP Payment Information File database, which was linked to the matching prescriber identifier in the PharmaNet file |

Abbreviations: CVD: Cardiovascular Diseases; COPD: Chronic Obstructive Pulmonary Disease; HCV: Hepatitis C Virus; ICD-9/9-CM/10-CA: International Classification Disease 9^th^ revision, 9^th^ revision clinical modification, 10^th^ revision Canada; DTP: Drug Treatment Program; STOP: Seek and Treat for Optimal Prevention of HIV/AIDS (STOP HIV/AIDS); MSP: Medical Services Plan Payment Information File; DAD: Discharge Abstract Database

**Databases:** Drug Treatment Program (DTP) was used to measure demographics, hepatitis C virus (HCV) diagnosis, and HIV-suppressed viral load. Discharge Abstract Database (DAD) and Medical Services Plan (MSP) were used to measure comorbidities of interest; PharmaNet data were used to identify all prescriptions using drug/product identification number (DIN/PIN).

**Episodic Case Definition:** Per BC Ministry of Health guidelines, mood and anxiety disorder, and depression were defined according to episodic prevalence case definition rather than cumulative prevalence. Accordingly, cases meet specified case definition criteria (*supplementary table 3*) to qualify in a given year and are then carried forward in all subsequent years with at least one condition-related code including diagnostic codes, procedure codes, or drugs in any specific year.

**References:**

Chronic Disease Information Working Group. BC Chronic Disease and Selected Procedure Case Definitions version 2016, last updated February 2018. <http://www.bccdc.ca/health-professionals/data-reports/chronic-disease-dashboard#Case--Definitions>

1. <http://www.bccdc.ca/resource-gallery/Documents/Chronic-Disease-Dashboard/mood-anxiety-disorders.pdf>

2. Yazdani K, Salters K, Shen T, et al. A 20-year population-based study of all-cause and cause-specific mortality among people with concurrent HIV and psychotic disorders. *AIDS (London, England).* 2022;36(13):1851-1860.

3. Tian TY, Zlateva I, Anderson DR. Using electronic health records data to identify patients with chronic pain in a primary care setting. J Am Med Inform Assoc. 2013;20(e2):e275-e280. doi:10.1136/amiajnl-2013-001856

4. <http://www.bccdc.ca/resource-gallery/Documents/Chronic-Disease-Dashboard/ischemic-heart-disease.pdf>

5. <http://www.bccdc.ca/resource-gallery/Documents/Chronic-Disease-Dashboard/chronic-obstructive-pulmonary-disease.pdf>

6. <http://www.bccdc.ca/resource-gallery/Documents/Chronic-Disease-Dashboard/diabetes-mellitus.pdf>

7. <http://www.bccdc.ca/resource-gallery/Documents/Chronic-Disease-Dashboard/osteoarthritis.pdf>

8. Nanditha NGA, Paiero A, Tafessu HM, et al. Excess burden of age-associated comorbidities among people living with HIV in British Columbia, Canada: a population-based cohort study. BMJ Open 2021;11:e041734. doi: 10.1136/bmjopen-2020-041734

9. BC Cancer Agency <http://www.bccancer.bc.ca/>

10. British Columbia Center for Excellence in HIV/AIDS. Drug Treatment Program. 2020; <http://bccfe.ca/drug-treatment-program>.

# **Appendix 5. Sample of PWH with OAT episodes initiation in 2008-2014**

**Supplementary Table 4. The sample characteristics of PWH with first OAT episodes in 2008-2014, categorized by first OAT prescription type**

|  | **Overall**  **(n=1,030)** | **First OAT Prescription^*^ Type in 2008-2014** | |  |
| --- | --- | --- | --- | --- |
| **Sample Characteristics** |  | **Methadone**  **(n=1,003)** | **Buprenorphine**  **(n=27)** | **p-value ^**^** |
| **Gender** **^a^**  Men  Women | 604 (58.6)  426 (41.3) | 590 (58.8)  413 (41.1) | 14 (51.8)  13 (48.1) | 0.55 |
| **Age,** years,  median (Q1, Q3) | 41 (34, 47) | 41 (34, 47) | 43 (34,51) | 0.19 |
| **Ever retained ^b^**  No  Yes  No Previous OAT | 227 (22.0)  444 (43.1)  359 (34.8) | 225 (22.4)  437 (43.5)  341 (34.0) | <5 **^c^**  5-10 (25-30) **^c^**  18 (66.6) | **0.00** |
| **Comorbidities (not mutually exclusive) ^d^** | | | | |
| Psychotic Disorders | 121 (11.7) | 115 (11.4) | 6 (22.2) | 0.11 |
| Mood & Anxiety Disorders | 154 (14.9) | 146 (14.5) | 8 (29.6) | **0.04** |
| Chronic Pain | 447 (43.4) | 431 (42.9) | 16 (59.2) | 0.11 |
| Hepatitis C Virus | 592 (57.4) | 571 (56.9) | 21 (77.7) | **0.03** |
| Cardiovascular Diseases | 39 (3.7) | 37 (3.6) | <5 | 0.27 |
| COPD | 45 (4.3) | 41 (4.0) | <5 | **0.02** |
| Diabetes Mellitus | 37 (3.5) | 37 (3.6) | <5 | 0.62 |
| Cancer | 20 (1.9) | 19 (1.8) | <5 | 0.41 |
| Osteoarthritis | 38 (3.6) | 34 (3.3) | <5 | **0.01** |
| Liver Diseases | 247 (23.9) | 235 (23.4) | 12 (44.4) | **0.01** |
| **Suppressed Viral Load**,  <200 copies/ml **^e^**  No  Yes  Unknown | 335 (32.5)  338 (32.8)  357 (34.6) | 322 (32.1)  328 (32.7)  353 (35.1) | 13 (48.1) **^c^**  10-15 (40-45) **^c^**  <5 | 0.53 |
| **Prescriber Type ^f^**  General Practitioner  Specialist Physicians  Community Medicine  Unknown | 855 (83.0)  51 (4.9)  66 (6.4)  58 (5.6) | 832 (82.9)  48 (4.7)  66 (6.5)  57 (5.6) | 23 (85.1)  <5 **^c^**  <5 **^c^**  <5 **^c^** | 0.14 |
| **Starting Dose**, g/ml, median (Q1, Q3) |  | 40 (30, 50) | 10 (5, 10) |  |
| **Time to therapeutic dose, days** (if achieved, n=386) **^g^** | 18 (9.36) | 18 (9, 36) | 12 (3, 36) | 0.44 |

All variables are measured at first OAT episodes during the study timeframe. The term "first OAT episode" refers to the treatment episode constructed according to the initial dispensing of OAT medication following the individual's entry into the HIV cohort. Any OAT episodes that were initiated before the entry into the HIV cohort and either continued or ended during the study period were excluded from the analysis. Unless stated otherwise, the values are expressed as n (%).

**^*^** The first OAT prescription type is according to the first OAT Rx in the first treatment episode.

**^**^** The p-value shows the comparison of variables among individuals with their first episodes of methadone, and buprenorphine/naloxone in 2008-2014.

**^a^** The variable "gender" encompasses both cisgender and transgender individuals who identify as men or women.

**^b^** Ever history of retention was assessed using a five-year lookback window. For each treatment episode, retention was assessed based on treatment duration, defined as no interruption in the prescribed doses for at least 12 months.

**^c^** Values have been censored and masked for privacy reasons.

**^d^** Comorbid conditions were defined using pertinent diagnostic codes (ICD- 9/9-CM/10-CA codes or BC-specific codes/non-ICD diagnostic doses) in MSP, DAD, or DINPINs in PharmaNet data. A detailed description of covariates appears in *Appendix 4*.

**^e^** The HIV viral load suppression was defined as suppressed if the values for two consecutive tests, 90 days apart were <200 copies /ml using the HIV DTP registry.

**^f^** The variable prescriber type was defined based on the “provider specialty” field in MSP.

**^g^** Measured across all “first” OAT episodes during the study (i.e., ≥ 60 mg for methadone, ≥ 12 mg for Buprenorphine/Naloxon)

Abbreviations: OAT: opioid agonist therapy; PWH: people with HIV; Q1: 25% interquartile; Q3: 75% interquartile; COPD: chronic obstructive pulmonary disease; DINPIN; Drug/Product Identification Number; ICD-9/9-CM/10-CA: International Classification Disease 9^th^ Revision /9^th^ Revision, Clinical Modification/ 10th Revision, Canada; MSP: Medical Services Plan; DAD: Discharge Abstract Database; DTP: Drug Treatment Program; MME: morphine milligram equivalent

# **Appendix 6. OAT Uptake, categorized by OAT medication type**

**Supplementary Table 5. Uptake of OAT among PWH between January 2008 and March 2020, overall, and stratified by OAT medication type**

| **Calendar Year** | **PWH in the cohort** | **Overall OAT Uptake, n (%)** | **Methadone n (%)** | **Bup/Nal**  **n (%)** | **SROM**  **n (%)** | **iOAT**  **n (%)** |
| --- | --- | --- | --- | --- | --- | --- |
| 2008 | 1116 | 338 (30.2) | 336 (30.1) | <5 |  |  |
| 2009 | 1179 | 371 (31.4) | 367 (31.1) | 5 (0.4) |  |  |
| 2010 | 1210 | 371 (30.6) | 370 (30.5) | 6 (0.5) |  |  |
| 2011 | 1239 | 372 (30.0) | 364 (29.3) | 14 (1.1) |  |  |
| 2012 | 1251 | 375 (29.9) | 360 (28.7) | 23 (1.8) |  |  |
| 2013 | 1264 | 388 (30.7) | 371 (29.3) | 31 (2.4) |  |  |
| 2014 | 1268 | 412 (32.4) | 385 (30.3) | 49 (3.8) |  |  |
| 2015 | 1287 | 458 (35.5) | 393 (30.5) | 84 (6.5) | 5 (0.3) | 13 (1.0) |
| 2016 | 1295 | 508 (39.2) | 391 (30.1) | 177 (13.6) | 11 (0.8) | 8 (0.6) |
| 2017 | 1257 | 518 (41.2) | 358 (28.4) | 161 (12.8) | 99 (7.8) | 30 (2.3) |
| 2018 | 1220 | 548 (44.9) | 347 (28.4) | 128 (10.4) | 163 (13.3) | 36 (2.3) |
| 2019 | 1165 | 535 (45.9) | 322 (27.6) | 115 (9.8) | 180 (15.4) | 35 (3.0) |
| 2020 | 1103 | 241 (21.8) | 149 (13.5) | 31 (2.8) | 63 (5.7) | 13 (1.1) |

**Note:** The above supplementary table represents the OAT that was initiated in each year calendar year during the study period. The information for the year 2020 is incomplete and concludes on March 31^st^.

Abbreviations: OAT: opioid agonist therapy; PWH: people living with HIV; Bup/Nal: buprenorphine/naloxone; SROM: slow-release oral morphine; iOAT: injectable OAT.

# **Appendix 7. OAT Characteristics, by OAT medication type among PWH in 2008-2020**

**Supplementary Table 6.1. Overall OAT characteristics**

| **Calendar Year** | **Total # of Episodes** | **Total # of Retained Episodes** | **Retention Rates** | **Median Duration of Total Episodes (Q1-Q3), days** |
| --- | --- | --- | --- | --- |
| 2008 | 741 | 151 | 20.38 | 56 (12-250) |
| 2009 | 794 | 145 | 18.26 | 46.5 (10-195) |
| 2010 | 804 | 132 | 16.42 | 43 (10-213.5) |
| 2011 | 863 | 139 | 16.11 | 42 (8-182) |
| 2012 | 916 | 124 | 13.54 | 33.5 (8-160.5) |
| 2013 | 967 | 124 | 12.82 | 38 (10-163) |
| 2014 | 1044 | 117 | 11.21 | 33 (8-125.5) |
| 2015 | 1157 | 143 | 12.36 | 29 (7-126) |
| 2016 | 1300 | 151 | 11.62 | 23 (7-94) |
| 2017 | 1428 | 136 | 9.52 | 22 (6-87) |
| 2018 | 1493 | 132 | 8.84 | 27 (7-102) |
| 2019 | 381 | 28 | 7.35 | 22 (7-80) |

**Supplementary Table 6.2. Methadone treatment characteristics**

| **Calendar Year** | **Total # of Episodes** | **# of Retained Episodes** | **Retention Rates** | **# of Episodes Achieved TD** | **Median Time to TD** |
| --- | --- | --- | --- | --- | --- |
| 2008 | 738 | 148 | 20.05 | 263 | 15 (7, 34) |
| 2009 | 789 | 145 | 18.38 | 299 | 15 (6, 35) |
| 2010 | 799 | 132 | 16.52 | 258 | 13.5 (7, 30) |
| 2011 | 848 | 135 | 15.92 | 266 | 14 (7, 31) |
| 2012 | 882 | 118 | 13.38 | 265 | 13 (7, 29) |
| 2013 | 913 | 118 | 12.92 | 274 | 14 (6, 30) |
| 2014 | 956 | 110 | 11.51 | 249 | 15 (7, 43) |
| 2015 | 979 | 125 | 12.77 | 299 | 12 (6, 28) |
| 2016 | 977 | 115 | 11.77 | 300 | 16 (7, 34) |
| 2017 | 1007 | 98 | 9.73 | 326 | 16 (7, 30) |
| 2018 | 876 | 83 | 9.47 | 303 | 14 (7, 27) |
| 2019 | 216 | 19 | 8.80 | 323 | 14 (7, 24) |

**Supplementary Table 6.3. Buprenorphine/Naloxone treatment characteristics**

| **Calendar Year** | **Total # of Episodes** | **# of Retained Episodes** | **Retention Rates** | **# of Episodes Achieved TD** | **Median Time to TD** |
| --- | --- | --- | --- | --- | --- |
| 2008 | 3 | 3 | 100.00 | 0 |  |
| 2009 | 5 | 0 | 0.00 | 0 |  |
| 2010 | 5 | 0 | 0.00 | 0 |  |
| 2011 | 15 | 4 | 26.67 | 3 | 41 (33, 48) |
| 2012 | 34 | 6 | 17.65 | 4 | 9.5 (5, 234) |
| 2013 | 54 | 6 | 11.11 | 13 | 19 (4, 36) |
| 2014 | 88 | 7 | 7.95 | 13 | 9 (2, 12) |
| 2015 | 158 | 8 | 5.06 | 28 | 11 (4, 28) |
| 2016 | 300 | 24 | 8.00 | 97 | 8 (3, 35) |
| 2017 | 249 | 13 | 5.22 | 86 | 7 (3, 19) |
| 2018 | 191 | 12 | 6.28 | 57 | 12 (6, 30) |
| 2019 | 54 | 3 | 5.56 | 35 | 11 (6, 28) |

**Supplementary Table 6.4. Slow-release oral morphine treatment characteristics**

| **Calendar Year** | **Total # of Episodes** | **# of Retained Episodes** | **Retention Rates** | **# of Episodes Achieved TD** | **Median Time to TD** |
| --- | --- | --- | --- | --- | --- |
| 2015 | 5 | 5 | 100.00 | 3 | 43 (4, 52) |
| 2016 | 11 | 11 | 100.00 | 9 | 43 (7, 47) |
| 2017 | 136 | 17 | 12.50 | 45 | 14 (5, 28) |
| 2018 | 367 | 30 | 8.17 | 136 | 6 (2.5, 15) |
| 2019 | 98 | 4 | 4.08 | 144 | 6 (2, 13) |

**Supplementary Table 6.5. Injectable OAT treatment characteristics**

| **Calendar Year** | **Total # of Episodes** | **# of Retained Episodes** | **Retention Rates** | **# of Episodes Achieved TD** | **Median Time to TD** |
| --- | --- | --- | --- | --- | --- |
| 2015 | 15 | 5 | 33.33 | 0 |  |
| 2016 | 12 | 1 | 8.33 | 2 | 1.5 (1, 2) |
| 2017 | 36 | 8 | 22.22 | 19 | 1 (1, 2) |
| 2018 | 59 | 7 | 11.86 | 21 | 1 (1, 2) |
| 2019 | 13 | 2 | 15.38 | 21 | 2 (1, 5) |

**Note:** Due to incomplete data for the year 2020 and individuals who received OAT in 2019 not completing the necessary look-forward time window for retention definition criteria, the trend test for retention rates does not incorporate data from 2019 and 2020.

**Overall trend:** There was a significant decrease in retention in 2008-2018 (p-value < .0001).

**Methadone trend:** There was a significant decline in retention in 2008-2018 (p-value <.0001).

**Bup/Nal trend:** There was a significant decline in retention in 2010-2018 (p=0.00).

**SROM trend:** There was no significant change in retention in 2015-2018 (p=0.09)

**iOAT trend:** There was no significant change in retention in 2015-2018 (p=0.42)

Abbreviations: OAT: opioid agonist therapy; PWH: people with HIV; TD: therapeutic dose; Q1: 25% interquartile; Q3: 75% interquartile;

# **Appendix 8. Univariate Model**

**Supplementary Table 7. Univariate Model and Initial Covariate Selection**

|  | **Unadjusted Model**  **OR (95% CI)** |
| --- | --- |
| **Time-Fixed Variables ^a^** | |
| **Gender**  Men [ref]  Women | 1.00  0.99 (0.80-1.23) |
| **Age** (per 10-year increase) | **1.63 (1.44-1.85)** |
| **HCV (lifetime)**  No [ref]  Yes | 1.00  1.02 (0.83-1.26) |
| **Ever Retained, using a 5-year LBW**  No  Yes  No OAT Before Entering | 1.00  **2.87 (2.14-3.85)**  **1.38 (1.03-1.86)** |
| **Time-varying Categorical Variables  ^b^** | |
| Total days on OAT during the study (per 365.25 days increase) | **1.10 (1.07-1.13)** |
| **OAT Type  ^b*^**  Methadone [ref]  Bup/Nal  SROM  iOAT | 1.00  **0.51 (0.42-0.63)**  0.94 (0.77-1.15)  1.31 (0.85-2.02) |
| **Mood & Anxiety Disorder**  No [ref]  Yes | 1.00  1.00 (0.84-1.18) |
| **Psychosis**  No [ref]  Yes | 1.00  **0.70 (0.51-0.96)** |
| **Chronic Pain**  No [ref]  Yes | 1.00  1.03 (0.83-1.27) |
| **CVD**  No [ref]  Yes | 1.00  1.22 (0.75-1.99) |
| **COPD**  No [ref]  Yes | 1.00  1.21 (0.68-2.12) |
| **Diabetes Mellitus**  No [ref]  Yes | **1.00**  **0.51 (0.28-0.91)** |
| **Cancer**  No [ref]  Yes | 1.00  0.60 (0.31-1.19) |
| **Osteoarthritis**  No [ref]  Yes | 1.00  1.04 (0.63-1.70) |
| **Liver Diseases**  No [ref]  Yes | 1.00  0.85 (0.66-1.09) |
| **HIV Viral Load suppression (copies/ml)**  Not Suppressed [ref]  Suppressed <200  Unknown | 1.00  **1.53 (1.30, 1.82)**  **1.45 (1.22-1.72)** |
| **Imputed HIV Viral Load suppression (copies/ml)**  Not Suppressed [ref]  Suppressed <200  Unknown | 1.00  **1.50 (1.29-1.74)**  1.34 (0.99-1.80) |
| **Therapeutic Dose ^d^** | |
| **Achieved the Therapeutic Dose**  No [ref]  Yes  Unknown | 1.00  **6.78 (5.70-8.07)**  **0.48 (0.06-3.59)** |
| **Interaction Term** | |
| HCV * Methadone  HCV * Bup/Nal  HCV* SROM  HCV * iOAT | 1.07 (0.84-1.35)  0.90 (0.61-1.34)  0.86 (0.50-1.47)  **3.21 (1.30-7.91)** |
| Liver Diseases * Methadone  Liver Diseases * Bup/Nal  Liver Diseases * SROM  Liver Diseases * iOAT | 0.78 (0.59-1.04)  0.64 (0.39-1.04)  1.19 (0.64-2.21)  1.33 (0.55-3.26) |

**Note:** If in the univariate model, the covariates did not differ significantly between retained and non-retained episodes, they were not used for selection by the model.

^a^ Time-fixed variables measured at first OAT during the study period

**^b^** Time-varying variables are measured at the beginning of each new OAT episode except for OAT type **^b*^** which was measured as of the end of each OAT episode

**^c^** Missing viral load values (291 out of 720) were imputed using the most recent data available within a year before OAT dispensation from the DTP registry.

**^d^** Assessed across all treatment episodes (i.e., ≥ 60 mg for methadone, ≥ 12 mg for Buprenorphine/Naloxone, ≥ 240 MME for SROM, 200 mg for iOAT). We lacked information on the drug strength for "66123367," which accounted for 25% of iOAT episodes. However, this didn't affect retention evaluation. We assumed a therapeutic dose based on other DINPINs. There were 0.71% episodes with unknown status, imputed as "yes" in the modeling stage.

Abbreviations: OAT: opioid agonist therapy; PWH: people living with HIV; Bup/Nal: Buprenorphine/Naloxone; OR: odds ratio; CI: confidence interval; Q1: 25% interquartile; Q3: 75% interquartile; HCV: hepatitis C virus; COPD: chronic obstructive pulmonary disease; CVD: cardiovascular diseases; LBW: look-back window.
